# Ultrasmooth, extremely deformable and shape recoverable Ag nanowire embedded transparent electrode

**DOI:** 10.1038/srep04788

**Published:** 2014-04-25

**Authors:** Sanggil Nam, Myungkwan Song, Dong-Ho Kim, Byungjin Cho, Hye Moon Lee, Jung-Dae Kwon, Sung-Gyu Park, Kee-Seok Nam, Yongsoo Jeong, Se-Hun Kwon, Yun Chang Park, Sung-Ho Jin, Jae-Wook Kang, Sungjin Jo, Chang Su Kim

**Affiliations:** 1 Advanced Functional Thin Films Department, Korea Institute of Materials Science (KIMS), Changwon 641-831, Republic of Korea; 2 School of Architectural, Civil, Environmental and Energy Engineering, Kyungpook National University, Daegu 702-701, Republic of Korea; 3 Center for Hybrid Interface Materials, School of Materials Science and Engineering, Pusan National University, Busan 609-735, Republic of Korea; 4 Measurement & Analysis Team, National Nanofab Center, Daejeon 305-806, Republic of Korea; 5 Department of Chemistry Education, Pusan National University, Busan 609-735, Republic of Korea; 6 Professional Graduate School of Flexible and Printable Electronics, Department of Flexible and Printable Electronics, Chonbuk National University, Jeonju 561-756, Republic of Korea; 7 School of Energy Engineering, Kyungpook National University, Daegu 702-701, Republic of Korea; 8 These authors contributed equally to this work.

## Abstract

Transparent electrodes have been widely used in electronic devices such as solar cells, displays, and touch screens. Highly flexible transparent electrodes are especially desired for the development of next generation flexible electronic devices. Although indium tin oxide (ITO) is the most commonly used material for the fabrication of transparent electrodes, its brittleness and growing cost limit its utility for flexible electronic devices. Therefore, the need for new transparent conductive materials with superior mechanical properties is clear and urgent. Ag nanowire (AgNW) has been attracting increasing attention because of its effective combination of electrical and optical properties. However, it still suffers from several drawbacks, including large surface roughness, instability against oxidation and moisture, and poor adhesion to substrates. These issues need to be addressed before wide spread use of metallic NW as transparent electrodes can be realized. In this study, we demonstrated the fabrication of a flexible transparent electrode with superior mechanical, electrical and optical properties by embedding a AgNW film into a transparent polymer matrix. This technique can produce electrodes with an ultrasmooth and extremely deformable transparent electrode that have sheet resistance and transmittance comparable to those of an ITO electrode.

Flexible electronic devices have attracted great attention due to their advantages over the devices fabricated by conventional bulk silicon technology. These advantages include excellent portability, conformal contact with curved surfaces, small weight, and human friendly interfaces^1^,^2^,^3^. Indium tin oxide (ITO) is the most commonly used material for the fabrication of transparent electrodes, however, it is not suitable for flexible electronic devices because it is brittle and easily cracks under mechanical stress^4^,^5^. Recently, several other candidates for flexible transparent electrodes, including carbon nanotubes^6^,^7^, graphene^8^,^9^, conducting polymers^10^,^11^, and metal nanowires^12^,^13^ have been widely investigated as potential replacements for ITO. Among those candidates, Ag nanowire (AgNW) is considered as one of the most promising materials for flexible transparent electrodes due to its excellent optical transparency, electrical conductivity, and mechanical flexibility^14^,^15^,^16^,^17^,^18^,^19^. However, because the rough surface of AgNW coating is likely to cause short circuits in the devices, its surface morphology posed a major challenge to its application in flexible electronic devices. The roughness of the deposited AgNW networks on a flat substrate is intrinsically large; the peak-to-peak roughness is more than twice the diameter of the wires, because of the random arrangement of networks through stacking of the wires^20^,^21^,^22^. Here, we demonstrate a novel and simple method for the fabrication of a ultrasmooth and extremely deformable flexible transparent electrode. The resultant AgNW embedded electrode exhibits a have high transparency and low sheet resistance those are comparable to ITO electrode.

## Results

### Fabrication of Ag nanowire embedded transparent electrode

**Figure 1:**
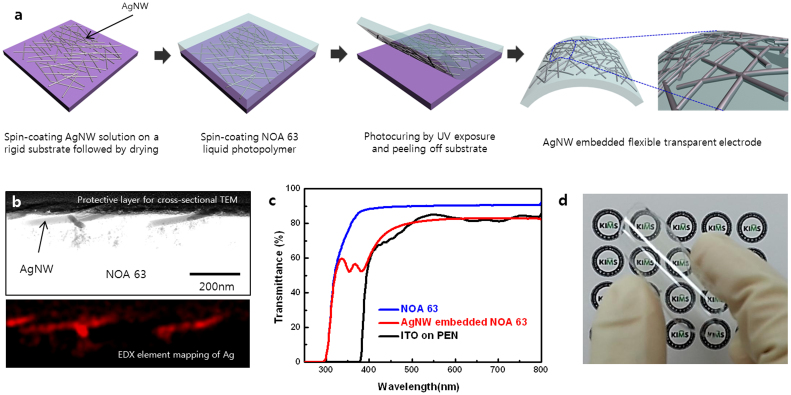
Fabrication process of the AgNW embedded flexible transparent electrode. (a) Schematic illustration of the fabrication process of AgNW embedded NOA 63 transparent electrode. (b) Cross-sectional TEM image and the corresponding elemental map of Ag in AgNW embedded NOA 63 transparent electrode. (c) Total transmittance spectra over the wavelengths 250–800 nm of NOA 63, AgNW embedded NOA 63, and ITO film on PEN. (d) Photograph of a fabricated AgNW embedded flexible transparent electrode (7 cm × 7 cm) illustrating their high transparency and flexibility.

The fabrication of the AgNW embedded transparent electrode followed a process schematically illustrated in Fig. 1a. As shown in the Supplementary Fig. S1, AgNW with an average diameter of 35 ± 5 nm and an average length of 25 ± 5 μm were employed in the fabrication. The AgNW dispersion obtained from NANOPYXIS was diluted with deionized water to a concentration of 3 mg/ml. It was then spin-coated on a pre-cleaned rigid substrate such as glass, Si wafer, or plastic, dried and annealed on a hotplate for 5 min at 100 °C to form a conductive AgNW coating. AgNW in the network were uniformly distributed and randomly oriented over the entire coating area. The electrical sheet resistance (R_s_) and optical transparency of the AgNW coating depended on the density of AgNW, which was controlled by spin-coating rate (rpm). Lower spin rates resulted in smaller deviations of the AgNW density and better uniformity of the coating (Supplementary Fig. S1), which led to smaller deviations of the R_s_ values. Further, we used a commercial ultraviolet (UV) curable polymer of Norland Optical Adhesive (NOA) 63 as the transparent substrate. NOA 63 is a clear, colorless and liquid photopolymer, whose polymer cross-linking will occur under exposer to UV light. The solid NOA 63 exhibits excellent optical transparency over a wide spectral range and superior mechanical flexibility; and it is commonly used in nanometer-scale contact printing and imprint lithography, or as a template-stripping photopolymer substrate^23^,^24^. The NOA 63 liquid photopolymer was spin-coated on the AgNW films and then cured by a 365 nm UV irradiation at room temperature for 10 min. Following the curing, the resulting AgNW embedded NOA 63 electrode with a thickness of about 100 μm was peeled off and cut into various sample sizes. Cross-sectional transmission electron microscope (TEM) image of an AgNW embedded NOA 63 electrode is shown in Fig. 1b. By employing an elemental mapping technique in TEM, the spatial elemental profile of Ag atom was obtained and the result is presented in Fig. 1b. These results clearly show that the AgNW network is well embedded in the NOA 63 substrate, implying a complete transfer of the nanowires from the original rigid substrate to NOA 63.

The optical transmittance and R_s_ of AgNW embedded NOA 63 electrode were compared with those of the sputtered ITO film on polyethylene naphthalate (PEN). The transmittance was measured including the substrates. As shown in Fig. 1c, the transmittance of AgNW embedded NOA 63 electrode approaches 82.3% at 550 nm wavelength with R_s_ value of 16 ± 1.75 Ω/sq, which were comparable to those of commercially available ITO electrodes on PEN (R_s_ of 15 ± 3.04 Ω/sq with the transmittance of 85.0% at 550 nm). Since most of the AgNW are embedded in the NOA 63 substrate, the low sheet resistance is a strong indication for the infiltration of NOA 63 in the pores of AgNW networks dispersed over the rigid substrate. It also suggest that the electrical contacts between the conductive AgNW networks are intact after formation of NOA 63 substrate through photopolymerization. Moreover, the tightening effect of strong bonding between the NOA 63 matrix and AgNW network allows the improvement of AgNW–AgNW contact, which resulted in a slightly smaller R_s_ than the original R_s_ (20 ± 2.87 Ω/sq) of the AgNW layer on rigid substrate. A remarkable difference in the transmittance between the two samples was the excellent optical transparency of AgNW embedded NOA 63 electrode in the UV region as compared with ITO film. The AgNW shows localized surface plasmon resonance bands in the wavelength region around 350 nm^25^. The AgNW embedded NOA 63 electrode showed 59.5% optical transmittance at 335 nm and its transmittance is almost flat across all the measured wavelength regions. Because the transmission in metal nanowire film is predominantly controlled by the density of the networks, in this case the sparse AgNW networks resulted in unchanged optical transmittance. In contrast, the transmittance of ITO film dropped rapidly at wavelength below 400 nm and displayed fluctuations over the visible light region. In principle, ITO shows high absorption in the UV light region^26^,^27^, making it difficult for practical use as a transparent electrode over the entire UV light region. Considering this fact, the great advantage of the AgNW embedded NOA 63 electrode is its broad optical transparency, allowing its use as transparent electrode over the entire UV light region. Figure 1d presents a photograph of AgNW embedded transparent electrode, behind which is the Korea Institute of Materials Science (KIMS) symbol mark being clearly visualized.

### Surface properties of AgNW embedded flexible transparent electrode

**Figure 2:**
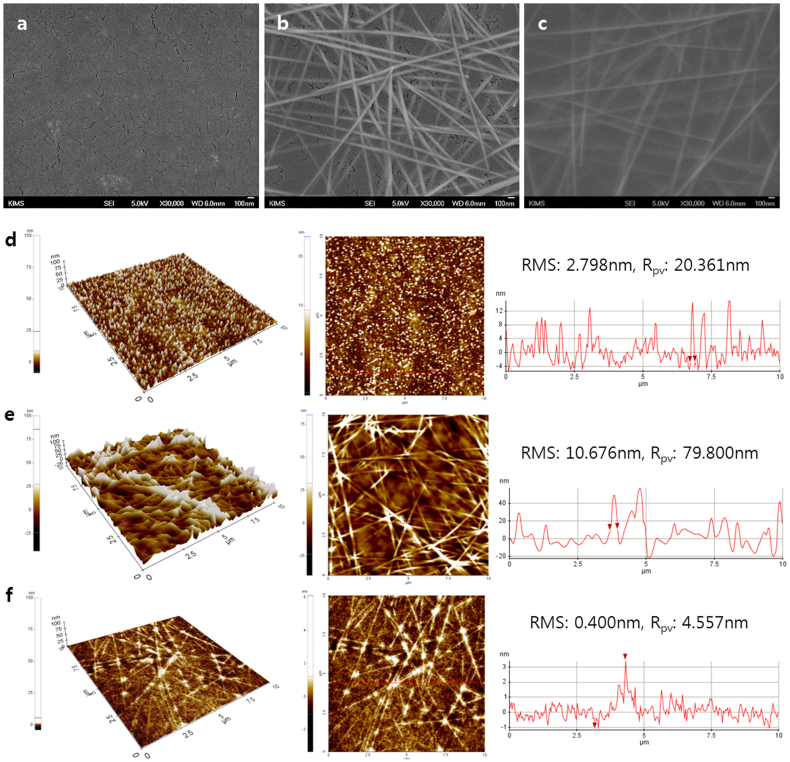
Surface properties of the AgNW embedded flexible transparent electrode. SEM images of the (a) ITO film on PEN, (b) AgNW coating on PEN, and (c) AgNW embedded NOA 63 electrode. Tapping mode AFM images with line scans as marked in the images and height line profiles of the (d) ITO on PEN, (e) AgNW on PEN, and (f) AgNW embedded NOA 63 (10 μm × 10 μm).

Figure 2 shows the images of scanning electron microscopy (SEM) and atomic force microscopy (AFM) of the ITO film on PEN substrate (Fig. 2a, d), AgNW coating on PEN substrate (Fig. 2b, e), and AgNW embedded NOA 63 electrode (Fig. 2c, e). The surface roughness of spin-coated AgNW layer on the rigid substrate was significantly larger than that of the commercially available ITO films. A root-mean-square surface roughness (RMS) of 10.676 nm and maximum peak-to-valley (R_pv_) range of 79.8 nm were noted for the AgNW layer. The presence of protruding nanowires leads to localized height elevations and this large surface fluctuation induces a higher possibility of electrical short circuits in the devices. It is obvious that, apart from transparency and conductivity, surface roughness is clearly another attribute to affects the compatibility of transparent electrodes with the device. In contrast, the AgNW embedded NOA 63 electrode displays an ultrasmooth surface with a RMS of 0.4 nm and R_pv_ of 4.557 nm, as can be seen from the high magnification AFM images in Supplementary Fig. S2. This ultrafine surface morphology indicates that the spin-coated NOA 63 liquid photopolymer well permeated the AgNW network, filled the holes in the networks, as well as the voids at the interface between AgNW and the rigid substrate.

### Extreme deformation of AgNW embedded flexible transparent electrode

**Figure 3:**
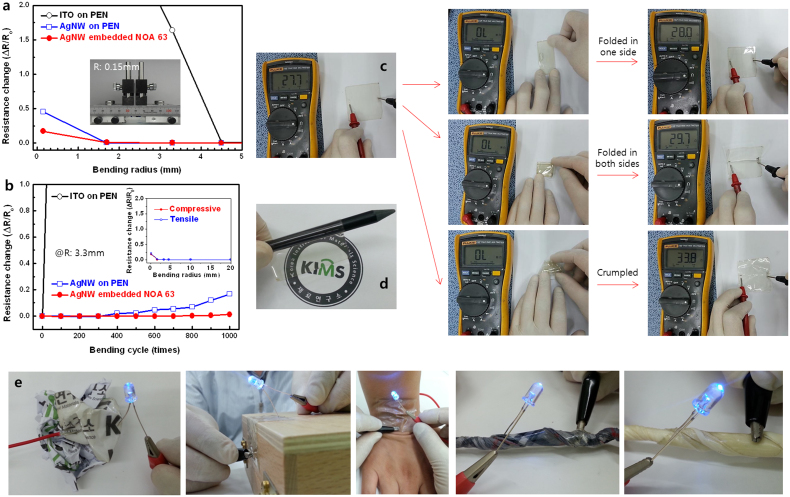
Demonstration of extreme deformation attainable with AgNW embedded flexible transparent electrode. (a) Relative change in the resistances of the ITO on PEN, AgNW on PEN, and AgNW embedded NOA 63 as a function of bending radius. The inset shows a photograph of a folded AgNW embedded flexible transparent electrode with a bending radius of 150 μm set for the bending test. (b) Relative change in the resistances of the ITO on PEN, AgNW on PEN, and AgNW embedded NOA 63 as a function of the number of bending cycles at the bending radius of 3.3 mm. The inset shows the resistance change with compressive and tensile stresses applied to the AgNW embedded NOA 63 electrode. (c) Photograph of AgNW embedded flexible transparent electrode that resembles a sheet of paper. The electrode is extremely deformable and can be repetitively folded and recover without deteriorating its conductivity. (d) Photograph of AgNW embedded flexible transparent electrode wrapped around a pencil with a radius of 3 mm. (e) Photographs of blue LED lamps mounted on AgNW embedded flexible transparent electrode transferred to various substrates, including crumpled paper, sharp wooded edge, human skin, stringed textile and rubber.

In addition to the excellent transparency and surface morphology, the AgNW embedded NOA 63 electrode possesses superior mechanical flexibility, which is a desirable attribute for the emerging flexible electronic devices. Figure 3a shows a comparison of the changes in the resistance of the electrodes, including ITO film on PEN substrate, AgNW coating on PEN substrate, and AgNW embedded NOA 63 electrode, as a function of bending radius. The presented values of the bending-induced compressive and tensile stresses in the films are the averages from ten samples for each type of electrode. The change in the resistance can be expressed as ΔR/R_0_, where ΔR is the actual change in the resistance after bending and R_0_ is the initial value. The AgNW coating on PEN showed fairly good mechanical flexibility with small ΔR/R_0_ value, even when a 2 mm bending radius was applied. In contrast, ITO film on PEN begun to crack when the bending radius approached to 5 mm, resulting in a sharp increase in the ΔR/R_0_ value. Since ITO is inherently brittle and can crack when exposed to a minute amount of strain. It is noteworthy that the ΔR/R_0_ value of the AgNW embedded NOA 63 electrode remained nearly constant (<0.25), even though the bending radius was 200 μm, which corresponded to a bending strain (ε) of 0.25%. Here ε = h_s_/(2R), where h_s_ is the substrate thickness^28^. The AgNW embedded NOA 63 electrode's electrical properties were more robust to both compressive and tensile stresses (Inset of Fig. 3b) when compared to Ag NWs on PEN. This is attributed to the strong bonding between the NOA 63 matrix and AgNW network, which could prevent the sliding from occurring at the interface under the extreme bending. Moreover, the AgNW embedded NOA 63 electrode showed excellent bending fatigue strength, as shown in Fig. 3b, in respect to a constant bending radius of at 3.3 mm. Even after the bending was repeated for 1000 times, the ΔR/R_0_ value of AgNW embedded NOA 63 electrode was almost the same as its original value, whereas that of the ITO film on PEN increased dramatically. We also observed minute change in the electrical resistance of the AgNW embedded NOA 63 electrode under the extreme conditions of folding and crumpling (Fig. 3c, Supplementary Fig. S3). Figure 3d shows an AgNW embedded flexible transparent electrode wrapping around a pencil with a radius of 3 mm; and Fig. 3e shows examples of AgNW embedded NOA 63 electrode successfully attached to different materials with uneven surfaces, including a crumpled paper, sharp wooden edge, human skin, stringed textile and rubber products, using a double sided transparent Scotch tape, which enables the conformal contact with various material surfaces, and makes the surface conductive without any surface treatment. Due to this excellent mechanical flexibility, the AgNW embedded NOA 63 electrode has a great potential to establish its applications as a transparent electrode platform in the flexible electronic devices.

### Thermal oxidation stability and shape memory property

**Figure 4:**
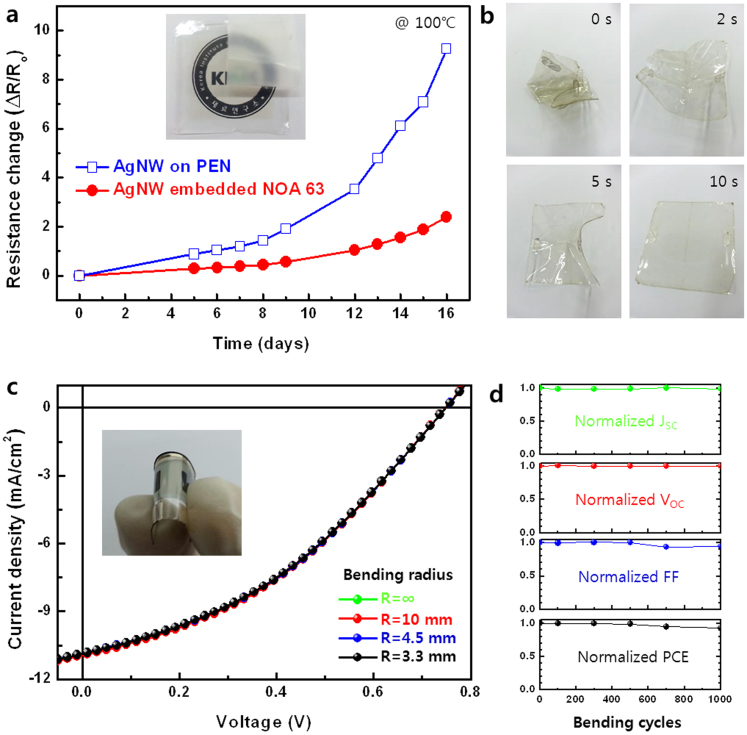
Shape memory property and performance of the flexible organic solar cells. (a) Relative change in the resistances of the AgNW on PEN and AgNW embedded NOA 63 as a function of the exposing time at 100°C and 70% RH for up to 16 days. The inset shows the mechanical adhesion test of AgNW embedded flexible transparent electrode after peeling off the 3 M Scotch tape. (b) A series of photographs showing the shape memory property of AgNW embedded flexible transparent electrode at 100°C. (c) Current density–voltage (J-V) characteristics of the flexible organic solar cell fabricated using AgNW embedded flexible transparent electrode as a function of the bending radius. The inset shows a photograph of the flexible organic solar cell device. (d) The measured J_sc_, V_oc_, FF, and PCE values of the flexible organic solar cell fabricated using AgNW embedded flexible transparent electrode as a function of the bending cycle at a bending radius of 3.3 mm, normalized to the initial value.

Furthermore, the AgNW coating on PEN and AgNW embedded NOA 63 electrode were placed in an environment of 100°C and 70% RH for 16 days to evaluate its thermal oxidation stability. Figure 4a shows the changes in the resistance after this thermal oxidation stability test. The ΔR/R_0_ value of AgNW on PEN increased dramatically when it was exposed to a high temperature and high humidity condition. It has been well known that AgNW can be easily oxidized when it is exposed to air. When AgNW is oxidized, the resistance of single nanowire and the junction will increase because of the formation of silver oxide on the surface of AgNW^29^,^30^. In contrast, the AgNW embedded NOA 63 electrode exhibited slightly increased ΔR/R_0_ value because the oxygen gas and moisture cannot permeate the NOA 63 matrix. Here the NOA 63 can act as a passivation layer to the fact that NOA63 is a mercapto ester-type solvent-less photopolymer^31^, allowing moderate UV curable cross-linking process, which resulted in lower density of statistical defects. A 3 M Scotch tape applied with finger pressure was unable to detach the AgNW from the AgNW embedded NOA 63 electrode (Inset of Fig. 4a) and the resistance of the electrode remained unchanged, thus suggesting strong adhesion.

Additionally, the AgNW embedded NOA 63 electrode showed excellent shape memory property. The electronic device failure as a result of mechanical fracture limits the lifetime and reliability of the devices, and leads to an increasing amount of electronic waste. Shape memory polymer has been explored that could help alleviate these problems^32^,^33^. The shape memory property of AgNW embedded NOA 63 electrode is exemplarily demonstrated in Fig. 4b and Supplementary Movie S1, where the crumpled sheet rapidly recovered to its original flat shape at 100°C within 10 s. After the recovery, the resistance of the AgNW embedded NOA 63 electrode was measured and was found similar to its original value. The intrinsic mechanism for the shape memory property of polymer is the freezing and activation of the long range motion of polymer chain segments below and above glass transition temperature (T_g_), respectively. The deformed electrodes therefore can reversibly relax back to the original shape when heated above T_g_ without the application of external tension or compression^34^.

## Discussion

Flexible organic solar cells were further fabricated by using the AgNW embedded NOA 63 electrode as described above to make them well suited for use in flexible electronic devices. Figure 4c shows the current density–voltage (*J*–*V*) characteristics of the devices as a function of bending radius under simulated AM 1.5 G illumination. The compressive stresses were delivered once every cycle and then the device performances were measured after the device was relaxed to back to planar shape. A blend of poly(4,8-bis-alkyloxybenzo(1,2-b:4,5-b′)dithiophene-2,6-diyl-alt-(alkyl thieno(3,4-b) thiophene-2-carboxylate)-2,6-diyl) (PBDTTT-C) and [6,6]-phenyl-C_61_-butyric acid methyl ester (PCBM) was used as the photoactive layer in the devices. The *J*–*V* characteristics of the device showed a Fill factor (FF) of 37.87%, a short circuit current density (J_sc_) of 10.92 mA/cm^2^, a open circuit voltage (V_oc_) of 0.74 V, and a power conversion efficiency (PCE) of 3.07%. The *J–V* characteristics of the devices remained almost the same even after the cell was bent with a radius of 3.3 mm. In addition, the device using the AgNW embedded NOA 63 electrode also exhibited excellent mechanical durability under repeated bending; almost no change in its *J*–*V* characteristics even after 1000 bending cycles using a 3.3 mm bending radius (Fig. 4d). In addition, *J-V* characteristics of the organic solar cells fabricated various bottom electrodes was shown in Supplementary Fig. S5. The device using the AgNW on PEN exhibited a significant shunt leakage current. The large roughness values of AgNW coating on rigid substrates can cause this device shorting. In the case of the device using the ITO electrode, the cell efficiency was higher than that of device using the AgNW embedded NOA 63 electrode. The reason for the slightly lower cell efficiency seen in the latter device was the small decreases in their fill factor (FF) values. The AgNW network contains some empty spaces between the nanowires, which limit the contact area with the top photoactive layer. This prevents the efficient collection of charge carriers by the AgNW network when the diffusion length of the charge carrier is shorter than the average void between the AgNW networks, despite the low sheet resistance. This lateral charge collection issue can be resolved by filling the voids with a conductive buffer layer^35^.

In summary, we have demonstrated that a flexible transparent electrode with superior mechanical, electrical and optical properties can be fabricated by embedding the AgNW film into the transparent NOA 63 matrix. This technique can produce electrodes with an ultrasmooth and extremely deformable transparent electrode that have sheet resistance and transmittance comparable to those of an ITO electrode. The unique shape memory property allowed the flexible transparent electrode to be formed into various shapes. This deformation was reversible and only induced minimal change to its original conductive property. This electrode is demonstrated to be suitable for the fabrication of flexible organic solar cells. We believe that the technique presented here will offer extended possibility of employing AgNW embedded electrode in the development of various efficient flexible electronic devices.

## Methods

### Fabrication of AgNW embedded flexible transparent electrode

The AgNW films were fabricated using the spin-coating method and were formed on a pre-cleaned rigid substrate such as glass, Si wafer, or plastic. An as-received dispersion containing AgNW (NANOPYXIS) was spin-coated for 40 s at speeds ranging from 600 to 3000 rpm. The dispersion was well shaken before being used in the spin-coating process. The formed Ag NW films were annealed at 100°C for 5 min on a hotplate. NOA 63 was spin-coated on the AgNW films at 500 rpm, and then cured with 365 nm UV irradiation at room temperature for 10 min. After the exposure, the resulting AgNW embedded NOA 63 with a thickness of about 100 μm was peeled off and cut into various sample sizes.

### Fabrication of flexible organic solar cell

The ZnO precursor was prepared by dissolving zinc acetate dihydrate (Zn(CH_3_COO)_2_·2H_2_O, Aldrich, 99.9%, 1.64 g) and ethanolamine (NH_2_CH_2_CH_2_OH, Aldrich, 99.5%, 0.5 g) in 2-methoxyethanol (CH_3_OCH_2_CH_2_OH, Aldrich, 99.8%, 10 g) under vigorous stirring at 60°C for 30 min to the hydrolysis reaction in air. The ZnO precursor solution was spin-coated on top of the AgNW embedded NOA 63 electrode. The films were annealed at 150°C for 10 min in air. The ZnO film thickness was approximately 40 nm. The ZnO-coated substrates were transferred into a nitrogen-filled glove box. A solution containing a mixture of PBDTTT-C:PCBM (10 mg:20 mg) was dissoved in 1,2-dichlorobenzene (1 mL). The PBDTTT-C, and PCBM used in this study were purchased from 1-material and Nano-C, respectively. The active layer was then spin-coated onto the ZnO-coated AgNW embedded NOA 63 electrode at 1000 rpm for 40 s after passing through a 0.20 μm PTFE syringe filter and the thickness of the active layer was about 100 nm. The PEDOT-PSS (Clevios 4083) diluted using isopropyl alcohol (IPA), with the ratio of PEDOT-PSS:IPA being 1:10, were deposited onto the active layer at 5000 rpm 60 s in a glove box. Finally, the Ag metal as top electrode was deposited through a shadow mask by thermal evaporation in a vacuum of about 5 × 10^−6^ Torr. The device area, defined through a shadow mask, was 0.38 cm^2^.

### Bending test

To investigate the mechanical stability, an in-house bending test system was designed and applied. The system consists of two contact interface to induce compressive stressed to the sample, where one is fixed and immobile, while the other is allowed to move laterally. Supplementary Fig. S4 shows the procedure followed in the bending tests for the flexible transparent electrode and organic solar cells.

## Author Contributions

C.S.K., D.H.K. and S.J. conceived and designed the research. S.N., M.S., B.C., H.M.L., J.D.K., S.G.P., K.S.N., Y.J., S.H.J. and J.W.K. participated in materials preparation, device fabrication and data interpretation. S.H.K. and Y.C.P. carried out the TEM measurements. C.S.K., D.H.K. and S.J. wrote the paper. C.S.K. supervised the project. All authors discussed the results and commented on the manuscript.

## Supplementary Material

Supplementary Information Supplementary information

Supplementary Information Supplementary Movie S1
