# Supplementary material for: Ultrasmooth, extremely deformable and shape recoverable Ag nanowire embedded transparent electrode
Source: Sci Rep. 2014 Apr 25;4:4788. doi: 10.1038/srep04788 (PMC3999473; doi:10.1038/srep04788)
Supplement: Supplementary Information [file srep04788-s1.doc]

**Supplementary information for**

**“Ultrasmooth, extremely deformable and shape recoverable Ag nanowire embedded transparent electrode”**

Sanggil Nam^1,2†^, Myungkwan Song^1†^, Dong-Ho Kim^1*^, Byungjin Cho^1^, Hye Moon Lee^1^, Jung-Dae Kwon^1^, Sung-Gyu Park^1^, Kee-Seok Nam^1^, Yongsoo Jeong^1^, Se-Hun Kwon^3^, Yun Chang Park^4^, Sung-Ho Jin^5^, Jae-Wook Kang^6^, Sungjin Jo^2,7*^ & Chang Su Kim^1*^

^1^Advanced Functional Thin Films Department, Korea Institute of Materials Science (KIMS), Changwon 641-831, Republic of Korea

^2^School of Architectural, Civil, Environmental and Energy Engineering, Kyungpook National University, Daegu 702-701, Republic of Korea

^3^National Core Research Center for Hybrid Materials Solution, Pusan National University, Busan 609-735, Republic of Korea

^4^Measurement & Analysis Team, National Nanofab Center, Daejeon 305-806, Republic of Korea

^5^Department of Chemistry Education and Interdisciplinary Program of Advanced Information and Display Materials, Pusan National University, Busan 609-735, Republic of Korea

^6^Professional Graduate School of Flexible and Printable Electronics, Department of Flexible and Printable Electronics, Chonbuk National University, Jeonju 561-756, Republic of Korea

^7^School of Energy Engineering, Kyungpook National University, Daegu 702-701, Republic of Korea

^†^These authors equally contributed to this paper.

^*^e-mail: [cskim1025@kims.re.kr](mailto:cskim1025@kims.re.kr); [dhkim2@kims.re.kr](mailto:dhkim2@kims.re.kr); [sungjin@knu.ac.kr](mailto:sungjin@knu.ac.kr)


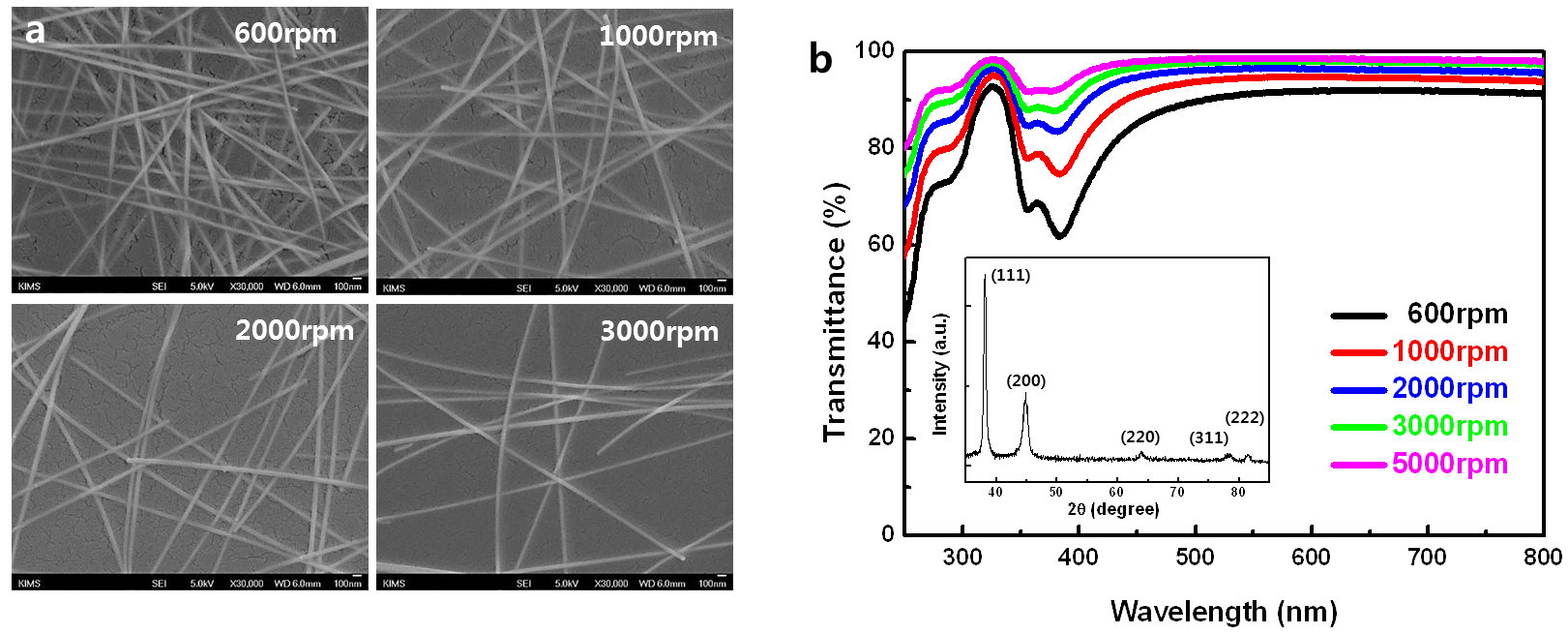
**Supplementary Figure S1.** (**a**) SEM images and (**b**) Total transmittance spectra of AgNW films at different spin-coating rates. The inset shows an X-ray diffraction pattern of the AgNW film.


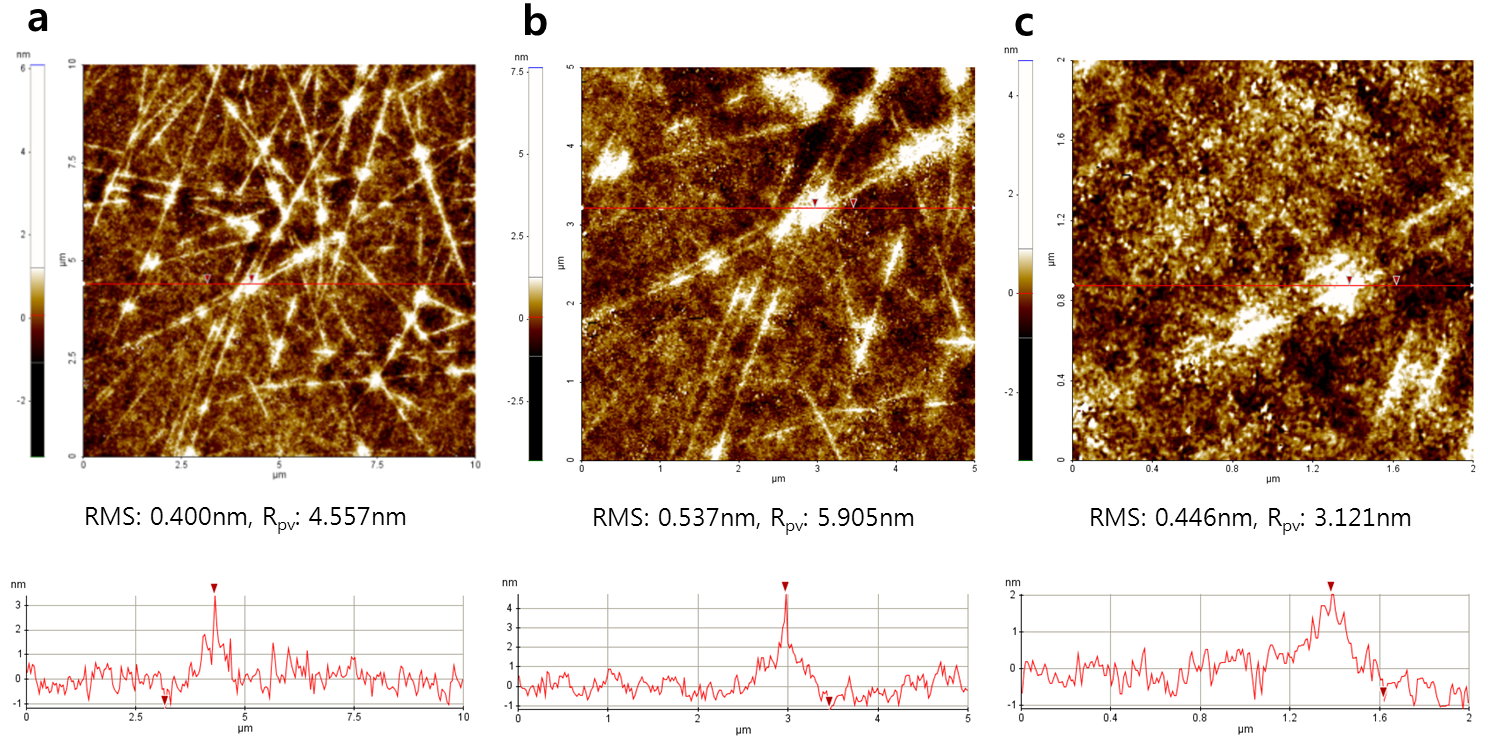
**Supplementary Figure S2.** Tapping-mode AFM images with line scans as marked in the images and line profiles of the AgNW embedded NOA 63 at scan are (**a**) 10 μm × 10 μm, (**b**) 5 μm × 5 μm, and (**c**) 2 μm × 2 μm.


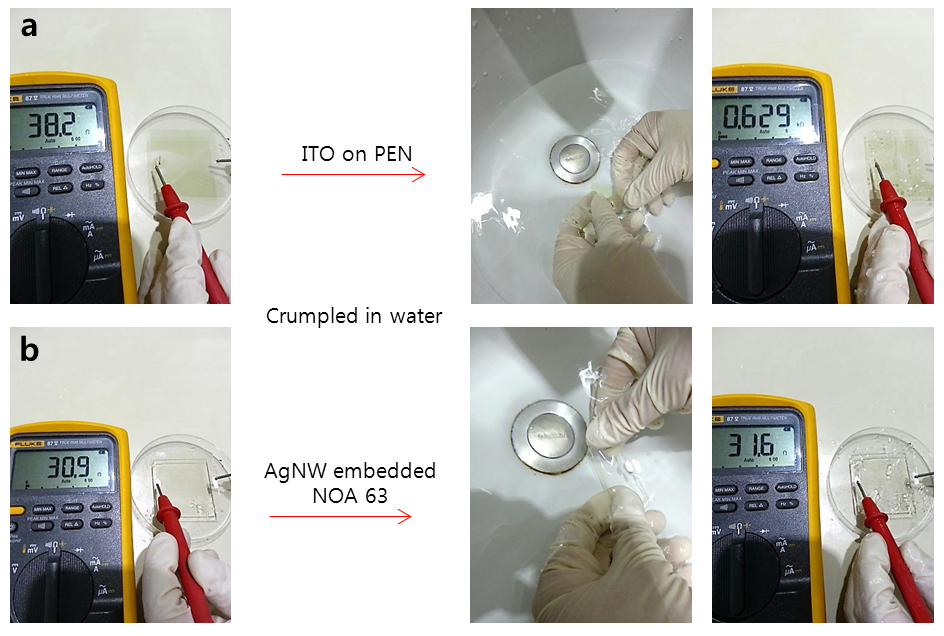


**Supplementary Figure S3.** Relative resistance changes in the ITO on PEN and AgNW embedded NOA 63 electrode crumpled in water. The optical photograph demonstrates that the AgNW embedded flexible transparent electrode can be even folded and crumpled in water.


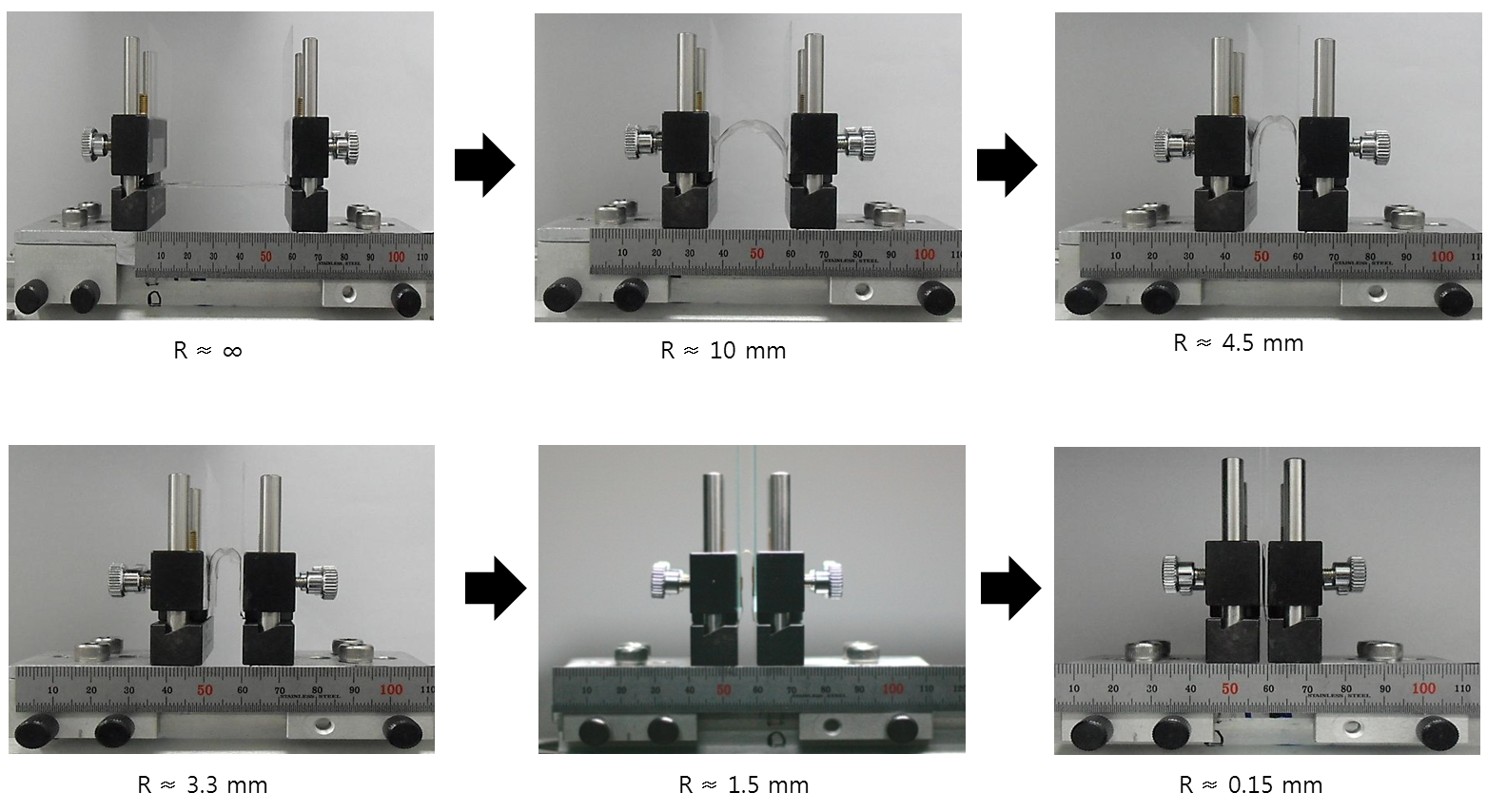
**Supplementary Figure S4.** Photographs of the bending test on a flexible transparent electrode and organic solar cells in progress using different bending radius.


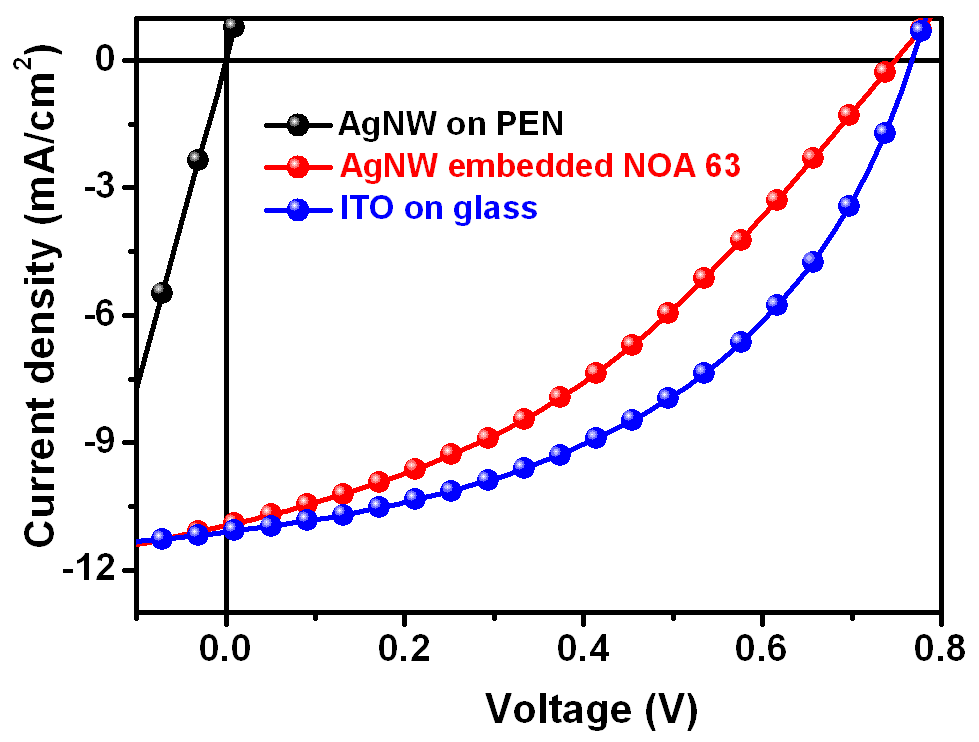


**Supplementary Figure S5.** Current density–voltage (J-V) characteristics of the organic solar cells fabricated with various bottom electrodes.
